# Are women’s breast cancer risk appraisals in line with updated clinical risk estimates communicated? Results from a UK Family History Risk and Prevention Clinic

**DOI:** 10.1158/1055-9965.EPI-24-0581

**Published:** 2024-12-02

**Authors:** Victoria G Woof, Anthony Howell, Lynne Fox, Lorna McWilliams, D Gareth R. Evans, David P French

**Affiliations:** 1 University of Manchester, Oxford Road, Manchester, M13 9PL, UK; 2 Family History Risk and Prevention Clinic, Prevent Breast Cancer Unit. Nightingale Centre, Southmoor Road, Wythenshawe Hospital, Manchester University NHS Foundation Trust, Manchester, M23 9QZ, UK

## Abstract

**Background:**

The incorporation of breast density and a polygenic risk score (PRS) into breast cancer risk prediction models can alter previously communicated risk estimates. Previous research finds that risk communication does not usually change personal risk appraisals. This study aimed to examine how women from the Family History Risk (FH-Risk) study appraise their breast cancer risk following communication of an updated risk estimate.

**Methods:**

In the FH-Risk study 323 women attended a consultation to receive an updated breast cancer risk estimate. A subset (n=190) completed a questionnaire, assessing their subjective breast cancer risk appraisals, satisfaction with the information provided and cancer related worry. One hundred and three were notified of a decrease risk, 34 an increase and 53 an unchanged risk.

**Results:**

Women’s subjective risk appraisals were in line with the updated risk estimates provided, with age, a PRS and breast density explaining most of the variance in these appraisals. Those notified of an increased risk demonstrated higher subjective risk perceptions compared to those whose risk remained unchanged or decreased.

**Conclusions:**

Women’s subjective breast cancer risk appraisals are amenable to change following updated risk feedback, with new information breast density and a PRS accepted and integrated into existing risk appraisals. Trust in the service, the analogies and visual communication strategies used may have positively influenced the integration of this new information.

**Impact:**

Further research is warranted to assess whether similar patterns emerge for other illnesses and in different clinical contexts to determine the best strategies for communicating updated risk estimates.

## Introduction

Breast cancer risk estimation in the UK is available for women with a strong family history of the disease (i.e. women with affected first or second-degree female relatives who meet the referral criteria). These women are referred via primary care to family history risk and prevention clinics (FHRPCs) and/or to genetic testing services for an assessment of pathogenic variants associated with breast cancer [1]. To calculate breast cancer risk in FHRPCs multifactorial risk prediction models are used, including the Tyrer-Cuzick (https://ems-trials.org/riskevaluator/) and CanRisk models [2,3]. These models include standard risk factors such as, age, family history, hormonal and reproductive factors (including age at menarche/menopause, parity and hormone medication use, for example hormone replacement therapy (HRT) and the oral contraceptive pill) and body mass index (BMI) to calculate risk. Health behaviours and diet (i.e. smoking and alcohol intake) are also incorporated into these models. Those found to be at an above-average (moderate) 10-year risk (5-7.99%) or at a high 10-year risk (>8%) are offered additional screening up to age 60 years, preventative medication and health behaviour change advice in line with the National Institute of Health and Clinical Excellence (NICE) clinical guidelines [1].

Recently, the inclusion of new strong independent risk factors, breast density (ratio of fibro-glandular tissue to fat) and a polygenic risk score (PRS; a calculation of single nucleotide polymorphisms (SNPs) related to hereditary breast cancer) has improved the validity and discriminatory capabilities of risk prediction models, including for the Tyrer-Cuzick and CanRisk models [4,5,6,7]. As the inclusion of these risk factors into risk prediction models results in more information risk estimates, as well as greater treatment stratification, it is plausible that these new risk factors will be incorporated into clinically settings more widely in the near future. Should this happen it is likely that previously counselled risk estimates provided in FHRPCs may change, potentially altering preventative management options for women.

The communication of new risk estimates, including new risk factor information, may be problematic as well as confusing for women. For instance, research to date suggests that following the provision of clinically-derived risk estimates, women’s breast cancer risk appraisals are typically not in line with the estimate provided, with some even distrusting the estimate, affecting their preventative management decisions [8,9,10]. A recent systematic review of the qualitative literature in this area suggested that women’s pre-existing expectations did not align with the clinical estimate provided and misperceptions about risk factors led to women feeling dubious about their risk results [9]. Similar findings were also found in a sample of women from the general population who received notification of an increased breast cancer risk estimate via population screening. Here it was found that personal experiences of those with breast cancer and being unable to personally identify with the risk factors contributing to their risk estimates (for example, health behaviours and family history) resulted in some women disbelieving their clinically-derived risk estimates, affecting their preventative management decisions and favouring their pre-existing subjective risk appraisals [11]. From this evidence it appears that women’s personal breast cancer risk appraisals are relatively stable and often remain unchanged by the communication of numerical clinical risk information.

As feedback regarding breast density and a PRS are not yet widely provided clinically in the UK, there is limited research on how women understand and internalise this new risk information. However, it has been highlighted that even after clear notification, women do not appear to understand the association between breast density and risk (medRxiv 2020.04.02.20048371). Additionally, PRS research has illustrated that how this risk factor is communicated varies between healthcare professionals, indicating the need for guidelines to support communication of such complex information [12]. Nevertheless, our findings from interviews with women who received notification of a change in their risk from the family history risk (FH-Risk) study found that they were accepting of and trusted the new risk estimates provided and understood the ‘gist’ of why their risk had changed by drawing on the breast density and PRS information communicated (Research Square rs.3.rs-3643438/v1). However, these findings were based on a small sub-sample (n=22) and it is therefore unclear how representative they are of all women who received updated risk feedback from the FH-Risk study.

Although healthcare professionals across other disease areas, such as diabetes and cardiovascular disease communicate clinically-derived risk estimates to patients regularly, there does not appear to be any research in this area which has assessed how updates or changes to risk estimates are understood and whether subjective risk appraisals alter as a result of this new information. As the inclusion of breast density and a PRS into breast cancer risk prediction models are improving their explanatory power, there is an opportunity to address this gap in the literature by assessing how women who require a recalculation of their risk view this update and whether they incorporate this new information into existing personal breast cancer risk appraisals. Thus the overall aim of the present study was to assess how women from the FH-Risk study appraised their breast cancer risk following a clinical update, which incorporated all currently known and validated risk factors. Specific objectives included:

1. To assess whether subjective risk ratings (perceived relative risk, defined as women’s perceptions of their 10-year risk of developing breast cancer compared to women of the same age) are associated with objective risk estimates (updated risk estimate (10-year)), and to identify which factors predict these subjective risk ratings, including standard risk factors, family history, a PRS and breast density.
2. To examine whether women perceive a change in their risk and whether perceptions of change are in line with changes in the objective risk estimates communicated.
3. To examine women’s satisfaction with updated risk information provided, as well as their cancer related worry.

## Materials and methods

### Design

The present study was a cross-sectional questionnaire study following communication of updated risk estimate information, which was communicated to women in risk consultations.

### Participants and setting

To be eligible for the present study, women had to have been patients at a North West England, UK FHRPC and received a summary letter of their updated breast cancer risk consultation from the FH-Risk study. Women were also required to have a negative test result for the 12 moderate and high penetrance genes tested. Women diagnosed with breast cancer after receiving their updated risk were excluded. Women who also took part in the interview sub-study (n=22) were also excluded (Research Square rs.3.rs-3643438/v1).

### Procedure

Women who took part in the original FH-Risk study from 2010 to 2012 [13,14] were invited in 2022/23 to receive an update on their breast cancer risk which included breast density and a PRS. The original FH-Risk study aimed to validated and improve breast cancer risk prediction models used in FHRPCs. The study population consisted of 954 women referred to the FHRPC between the years 1990 and 2012, where their initial risk of developing breast cancer was calculated at first referral. Between 1990 and 2004 lifetime risk was calculated by using modified Claus tables to include endocrine risk factors [15]. From 2004 risk was calculated using the Tyrer-Cuzick model [2]. Comparison of the two methods across 8,824 women attending the FHRPC was performed and indicated no significant difference between the two models of risk [16]. During their participation in the original FH-Risk study women gave their consent for DNA testing (used for SNP analysis) and for the calculation of their breast density. However, women only received partial feedback regarding SNPs and breast density as these risk factors had not been fully validated into risk prediction models at the time, therefore personalised feedback was not possible as more research was required.

However, now that breast density and a PRS have been validated and included in risk predication models (i.e. the Tyrer-Cuzick and CanRisk models) it was possible in 2022/23 for women who had participated in the original FH-Risk study to be given the opportunity to be notified of their updated breast cancer risk estimate. Three-hundred and twenty-three women took up this offer. Women received a telephone consultation where their updated risk was discussed, although this consultation was in person for a small proportion. The same consultant provided all risk feedback. After each consultation, women received a letter which included a summary of what was discussed and an image indicating their breast density (supplementary materials 1). To aid understanding of their PRS, the consultant provided women with an accessible analogy during consultations (supplementary materials 1). Updated risk feedback was based on the mean 10-year risk score of the Tyrer-Cuzick (https://ems-trials.org/riskevaluator/) [2,17] and CanRisk [3] models, which incorporated standard risks factors, as well as new risk factors breast density (measured using BIRADS™) and a PRS (a calculation based on 313 genetic variants associated with breast cancer known as single nucleotide polymorphisms (SNP313)). It was decided clinically to provide women with the mean score of these two models, given that CanRisk is a more conservative measure of risk compared to the Tyrer-Cuzick. Although both models have demonstrated validity, the literature remains unclear on which model is superior in the present context. Table 1 provides the mean 10-year risk estimates for the models for both the present sample and the overall FH-Risk sample. Women also received feedback on 12 moderate and high penetrance genes associated with breast cancer. For some women a change in risk resulted in a change in their risk management, including eligibility for additional mammographic screening and preventative medication.

For the present study, women who had been notified of their updated breast cancer risk and received their summary letter post consultation were invited to complete a paper questionnaire via post. Reminder invites were distributed 3-weeks after the initial study invite. Women received questionnaires as long as 15.4 months after their updated breast cancer risk notification and as little as 2.3 months after (mean=8.5 months;SD3.60).

### Measures

The following measures were used in the questionnaire (supplementary materials 1):

*Perceived relative risk of developing breast cancer (relative risk ratings):* a single-item asked women to rate their breast cancer risk in the next 10 years, compared to other women their age. Response options: *much higher, a bit higher, about the same, a bit lower, much lower* [18].

*Perceived absolute risk of developing breast cancer:* a single-item asked women to describe their percentage risk of developing breast cancer in their lifetime. Response options: *30% or greater (1 in 3), between 17% and 29.9% (1 in 4-6)* and *less than 17% (less than 1 in 6)*. Percentage brackets were based on the National Institute of Health and Clinical Excellence (NICE) clinical guidelines for familial breast cancer [1].

*Perceived change in risk:* a single-item asked women to rate how much their risk had changed following their updated risk consultation. Response options: *increased a lot, increased a little, stayed the same, decreased a little* and *decreased a lot*. An item was also provided if women felt that they did not know what their risk status was.

*Knowledge about breast cancer risk factors included in updated risk estimates:* six-items asked women what risk factors they believed were included in their updated risk estimates that were not included to calculate their initial risk estimates.

*Satisfaction with updated risk information:* four-items from a published scale [19], with seven response options ranging from, *strongly agree* to *strongly disagree (α = 0.90)*.

*Breast cancer worry:* assessed using a single-item from the Lerman Cancer Worry Scale [20]: “how often do you worry about developing breast cancer?” Response options: *never, rarely, sometimes* and *almost all of the time*.

*An open response box* was included to encourage women to mention anything that was important to them that they felt had not been covered in the questionnaire.

*Clinical information* was obtained from the FH-Risk study database with consent from the women participating (table 2 note).

### Data analysis

An a priori analysis plan was registered on the Open Science Framework (https://osf.io/ryn8p/). Data were analysed in IMB SPSS Statistics (version 29; RRID: SCR_016479).

Pearson’s correlation coefficient was performed to determine the relationship between the updated risk estimate and women’s relative risk ratings.

To examine the predictors of women’s relative risk ratings, a hierarchical multiple regression was conducted, with three blocks of variables: (a) family history only, (b) family history and standard risk factors and (c) family history, standard risk factors, a PRS and breast density. Sensitivity analysis was also performed to determine whether length of time between risk consultation appointments and completion of the questionnaire influenced women’s risk appraisals.

A one-way ANOVA was performed to examine whether women perceived a change in their risk following notification of their updated risk estimates.

An illustrative comparison of means was carried out to determine whether satisfaction and cancer worry scores in the present study were elevated. As we had no baseline or control group to act as comparator, we compared the scores to a comparable sample of women from the Breast Cancer Predict (BC-Predict) study. In the BC-Predict study women were invited as part of population screening to receive their 10-year risk of developing breast cancer which included standard risk factors, breast density and for some a PRS [21]. Satisfaction and cancer worry scores in the present study were compared to scores calculated at 6-months follow-up in the BC-Predict study.

### Ethics approval and consent to participate

Ethical approval was granted by HSC REC A ethics committee (ref: 21/NI/0130) and received HRA approval. The study was carried out in accordance with Good Clinical Practice guidelines and the Declaration of Helsinki, with all women providing written informed consent.

## Results

### Participating sample

Of the 301 women from the FH-Risk study who were eligible to participate in the questionnaire study, n=190 completed the questionnaire (63.1%) and were aged between 46-84 years (mean=63.00; SD7.05). This sample of women was representative of the wider FH-Risk study (n=323) sample where women were aged between 42-84 years (mean=61.16, SD7.63). Age for the present study was derived from the time of re-enrolment in the main FH-Risk study as this was used to calculate women’s updated risk estimates. All women identified as either White or White British, which is reflective of the wider FH-Risk study sample. Index of multiple deprivation (IMD) (http://imd-by-postcode.opendatacommunities.org/imd/2019) deciles calculated using residential postcode data ranged from one to ten (one indicating most deprived and ten least deprived), with a mean score of six. The average deprivation score for the wider FH-Risk sample was also six. Education and employment status data were not collected. In the wider FH-Risk sample (n=323), 162 (50.1%) women experienced a decrease in their risk by one or more NICE categories, 115 (35.6%) were notified that their risk was unchanged and 46 (14.2%) were told that their risk had increased by one or more NICE categories [1]. Of the 190 women who completed the questionnaire in the present study, 103 (54.2%) were notified that their risk had decreased by one or more NICE categories, 53 (27.9%) that their risk was unchanged and 34 (17.9%) that their risk had increased by one or more NICE categories.

### Outcomes

There was a large positive relationship between updated risk estimates and women’s relative risk ratings (r=.569, n=188, p<0.001), with higher relative risk ratings associated with higher updated risk estimates.

Family history did not significantly predict relative risk ratings. A model which additionally included standard risk factors explained 5.1% of the variance in women’s comparative risk ratings, with age significantly associated (*β*=-.229). Model three which added PRS and breast density scores was statistically significant and explained an additional 24.3% of the variance in women’s relative risk ratings, with age (*β*=-.215), a PRS (*β*=.289) and breast density (*β*=.453) significantly associated (table 2). These findings were not affected in a sensitivity analysis to test for the effect of level of deprivation and length of time between risk notification and questionnaire completion on women’s relative risk ratings.

There was an effect of updated risk estimates on women’s perceptions of a change in their risk (F(2,181)=18.78,p<0.001), with women who experienced an increase having significantly higher risk appraisals (3.53, 1.21, p<0.001) than women who experienced a decrease (2.21, 1.02, p<0.001) or no change (2.65, 1.15, p=0.001). There was no effect between women whose risk decreased and those who experienced no change (p=0.053).

Mean satisfaction scores (M=6.20; SD=0.78) and mean cancer worry scores (M=2.63; SD=0.66) in the present study were similar to the BC-Predict sample of women (M=6.40; SD=0.65; M=2.28; SD=0.68 respectively), with cancer worry scores not reaching clinical levels of concern.

There was variation in the extent to which women were aware of which variables were included in their initial and updated risk estimates (table 3).

## Discussion

This study has shown that women’s subjective breast cancer risk appraisals reflect the clinical updated risk estimate provided, with ratings of risk increasing in line with this estimate. Breast density and a PRS were found to be significant predictors of women’s relative risk ratings, together with age. Overall satisfaction and cancer worry scores in the FH-Risk cohort were comparable to those of women who received 10-year risk estimates in the BC-Predict study.

### What this study adds to the literature

This study provides new evidence that women’s personal breast cancer risk appraisals can align with the clinically-derived risk estimate communicated. This congruence is in contrast to a wealth of literature showing that even after a breast cancer risk estimate is communicated, women still hold inaccurate appraisals of their risk [8,9,10]. Findings from qualitative research we conducted with a sub-sample of the FH-Risk cohort provides some evidence for why this may be the case. Here it is suggested that women’s longstanding engagement with the service, where they have received positive and consistent quality care may influence the trust they have in the updated risk estimate, enabling them to develop a new understanding of their risk (doi.org/10.21203/rs.3.rs-3643438/v1). Compared to those who received their risk estimates via letter in the BC-Predict study [11], women in the present study could be described as more informed about breast cancer risk in general, due to their annual engagements with the FHRPC and thus having more opportunities to discuss their risk with a healthcare professional.

To our knowledge this is the first study to assess which objective breast cancer risk factors influence subjective risk appraisals. Previous qualitative research suggests that family history is a significant predictor of women’s subjective risk appraisals [9,11]. However, in the present study, age, a PRS and breast density were the only risk factors found to be significant. Breast density and a PRS being significant predictors is probably due to risk consultations focussing on the contribution of these new risk factors to women’s updated risk estimates. Specially, the analogy provided in consultations to aid understanding of complex PRS information and the visual representation of breast density in follow-up letters may have focussed women’s attention toward these risk factors. These findings illustrate that women understood the contribution these new risk factors made to their risk and were able to integrate this new information into their pre-existing risk appraisals, illustrating, in opposition to previous literature [9,10,11], that subjective breast cancer risk appraisals are adaptable.

### Strengths and limitations

In contrast to the extant literature, the findings from this study demonstrates new evidence that subjective appraisals of breast cancer risk are in line with updated risk estimates communicated clinically, with women integrating their knowledge of new risk factors into personal appraisals of their risk to inform new understandings. Furthermore, the sub-sample of women who completed the questionnaire from the FH-Risk cohort were representative of the wider cohort as a whole in regards to their age, ethnicity, mean risk estimates and mean deprivation scores. However, the study is not without its limitations. The time between the completion of the questionnaire and clinical consultations varied, which could have affected women’s recall of their risk estimates. Although when accounting for this, this factor did not appear to affect results. In addition, the FH-Risk sample was ethnically homogenous (white women) and details of women’s education status was not recorded as part of this study. It is possible that different findings would obtain with women of different ethnicity and level of education. Furthermore, the analysis presented would have been more informative had pre-disclosed subjective risk appraisals been collected, to allow comparisons of these to appraisals measured after an updated risk estimate had been communicated to assess for change. Finally, it is important to note that the study population was highly selective. It included only women from one UK FHRPC who participated in the FH-Risk studies and who were/had been receiving regular breast cancer risk-related care and advice. Therefore, the generalisability of the results is unclear.

### Implications for practice

This study illustrates that it is possible for women to obtain accurate subjective risk appraisals following the communication of an updated breast cancer risk estimate in clinical settings, as well as cancer related worry not reaching clinical levels of concern following this. These findings are in contrast to the present literature which states that even after genetic counselling sessions, women’s risk appraisals still do not align with the clinical estimate, despite modest improvements [8]. Instead this study has shown that updated risk estimates and information about breast density and a PRS can be understood and accepted by women if communicated effectively, with analogies and visual representations of risk factors, as well as women’s longstanding positive relationship with the clinic resulting in higher levels of trust in the information provided.

### Implications for research

The updated risk estimates provided in this study were communicated from a FHRPC characterised as a centre of excellence [22], with women encountering the same clinician throughout their service engagement. Similar studies should be conducted in other FHRPCs as well as non-specialist centres, with delivery of updated risk estimates provided by a variety of healthcare professionals to ascertain any differences in risk appraisals and to establish optimal communication. Furthermore, the sample obtained in the present study were majority White and living in areas of relative low deprivation. As research is ongoing to validate breast cancer risk prediction models across different ethnic groups [23], future research should consider how changes in risk are understood and experienced in more diverse populations of women.

A factor which was not examined in the present study was women’s views toward their eligibility for preventative management. However our findings from interviews with the FH-Risk cohort highlighted that women who were told that they were ineligible for annual screening had reservations about the effectiveness of population screening programmes and the safety of attending every three years (Research Square rs.3.rs-3643438/v1). Future research however is still warranted to explore the psychological impact of receiving a change in risk which may influence access to early detection and preventive management services. Additionally, assessing the relationship between subjective risk appraisals and the uptake of preventative management services in this context would also be worthwhile. This is especially important considering that the current literature indicates that providing a clinically-derived breast cancer risk estimate does not, in the majority of cases, lead to preventative medication use [9, 11, 24] or changes in health behaviours among those with a family history of the disease [9, 25].

Finally, this study has shown that women did take into account breast density and PRS information when forming appraisals of their breast cancer risk. However, some of the variance in women’s appraisals remained unexplained. Therefore, future research should consider under which circumstances more (or less) accurate risk appraisals are formed.

## Conclusion

This study illustrates that women can form accurate appraisals of their breast cancer risk following communication of their updated risk estimates. Given this, further research is needed to assess whether similar results are seen in other clinical settings and FHRPCs. This observational work will help determine the most effective ways of communicating updated risk estimates, which result in accurate personal risk appraisals and informed decisions about risk management.

## Supplementary Material

Supplementary Materials

## Figures and Tables

**Table 1:** Mean risk scores for the Tyrer-Cuzick and CanRisk models with the inclusion of breast density (BD) and a PRS for the FH-Risk questionnaire cohort (n=190) and overall FH-Risk cohort (n=323).

	Mean TC (SD) Questionnaire Sample (n=190)	Mean CR (SD) Questionnaire Sample (n=190)	Mean TC (SD) FH-Risk sample (n=323)	Mean CR (SD) FH-Risk sample (n=323)
Standard risk factors*	7.83 (2.39)	6.68 (2.26)	7.65 (2.51)	6.73 (2.35)
+BD	7.17 (3.32)	5.61 (2.54)	7.21 (3.63)	5.72 (2.53)
+PRS	8.01 (4.03)	6.54 (2.54)	8.12 (4.47)	6.59 (2.51)
+BD & PRS	6.99 (4.69)	4.71 (2.51)	7.04 (4.82)	4.88 (2.62)

* Family history, age, age at menarche, age at first full term pregnancy, parity, age at menopause, BMI

**Table 2:** Hierarchical multiple regression analysis for predicting women’s relative risk ratings from family history (FH), age, BMI, age at menarche, age at full-term pregnancy, parity, age at menopause, a PRS and breast density (BD).

Predictor	Initial *b [95% CI*^a^*]*	*Final β [95% CI* ^b^ *]*	sr^2c^	Adjusted R^2^ (*R^2^*_*adj*_)	Increased R^2^
**Model 1**				-.003	-.003
Family history (FH)	.062 *[-.103, .227]*	.056 [-.092, .203]	.056		
**Model 2**				.051*	.054*
FH	.083 *[-.086, .253]*	.075 [-.077, .227]	.074		
Age	-.039 *[-.064, -.014]*	-.229* [-.398, -.087]	-.229		
Age at menarche	-.103 *[-.215, .010]*	-.135 [-.283, .014]	-.126		
Age at full-term pregnancy	.007 *[-.011, .025]*	.078 [-.113, .272]	.062		
Parity	-.147 *[-.338, .044]*	-.144 [-.336, .044]	-.115		
Age at menopause	.017 *[-.021, .056]*	.066 [-.081, .213]	.068		
BMI	-.018 *[-.048, .012]*	-.091 [-.248, .060]	-.092		
**Model 3**				.294**	.243**
FH	.037 *[-.112, .185]*	.033 [-.100, .165]	.038		
Age	-.037 *[-.058, -.015]*	-.215** [-.363, -.094]	-.249		
Age at menarche	-.076 *[-.173, .022]*	-.099 [-.228, .029]	-.117		
Age at full-term pregnancy	.000 *[-.015, .016]*	.005 [-.162, .172]	.004		
Parity	-.106 *[-.271, .060]*	-.104 [-.269, .060]	-.096		
Age at menopause	.019 *[-.015, .052]*	.071 [-.056, .198]	.085		
BMI	.023 *[-.007, .053]*	.112 [-.039, .272]	.113		
PRS	.712 *[.397, 1.027]*	.289** [.162, .418]	.325		
BD	.545 *[.369, .720]*	.453** [.317, .618]	.426		

a *CI = Confidence Interval for b;*

b *CI = Confidence interval for β;*

c *sr^2^ = semi-partial correlation squared;*

* *p<0.05, p<0.001*.

*Note: Clinical information obtained from the FHRPC included, age, updated breast cancer risk, family history (i.e. first and second degree relatives affected by breast and/or ovarian cancer, including age at diagnosis and age at death (if applicable), age at menarche, parity, age at first live pregnancy, age at menopause and Body Mass Index (BMI)*

**Table 3:** Summary of women’s knowledge of the key risk factors included in their updated breast cancer risk estimates which were not included in their initial risk estimates received from the FHRPC.

Risk factors	Items (%) women understood to be included in their risk estimates at each stage (initial/updated estimates)			
	New risk factor	Included before	Not a factor included in my updated risk	Not sure
Age	12.5	59.2	11.4	16.8
Breast density	36.6	41.4	8.6	13.4
Family history	4.3	86.6	7.5	1.6
Breast cancer gene testing	22.5	45.5	13.9	15.0
Body mass index (BMI)	11.2	59.9	13.9	15.0
Single Nucleotide Polymorphisms (SNPs)	40.1	9.1	9.6	41.2

## Data Availability

The data generated in this study are publicly available in the Open Science Framework (https://osf.io/ryn8p/).
