# Supplementary material for: Are women’s breast cancer risk appraisals in line with updated clinical risk estimates communicated? Results from a UK Family History Risk and Prevention Clinic
Source: Cancer Epidemiol Biomarkers Prev. Author manuscript; Available in PMC 2024 Oct 29. (PMC7616752; doi:10.1158/1055-9965.EPI-24-0581)
Supplement: Supplementary Materials [file EMS199365-supplement-Supplementary_Materials.pdf]

Supplementary Materials 1

Updated risk consultation summary letter

Dear

Re: Exploring the experience of receiving updated breast cancer risk estimates in women with a family history of breast cancer: communicating a revised risk estimate

Thank you for taking the time to attend your risk consultation appointment about the reassessment of your breast cancer risk. The aim of the conversation was to let you know how your risk may have changed and whether this alters our advice about screening and prevention. This letter summarises what we spoke about.

Your given risk when you first attended the clinic was a 40% chance of developing breast cancer. This risk was estimated from the standard models we used at that time and using your family history, hormonal and lifestyle factors.

As we discussed, your risk may change with the passage of time but may also become more accurate, when we include factors like your breast density (the ‘whiteness’ of the mammogram) and DNA tests (SNP score and gene test).

Your breast density on your latest mammogram was recorded as “C”, moderate density and is pointed out on the diagram below. This degree of density increases your risk.

Your breast density

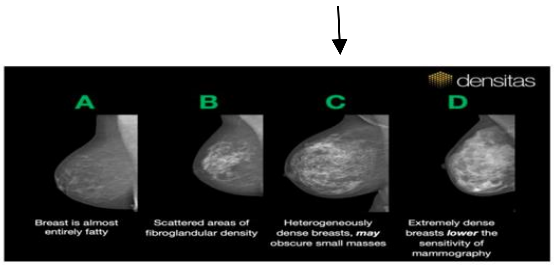

Your SNP score is 2.1 (average 1.0) which increases your risk.

Your test for 12 genes related to breast cancer and 21 to other cancers is negative.

Overall

We put all this information above together to get an indication of the change in your risk since your first visit to the clinic. Overall, your risk has increased.

In the next 10 years your risk of developing breast cancer is 16.8% compared with 3.1% in the general population.

During your lifetime your risk is 42% compared with 10.8% in the general population. Thus, you have about four times the population risk.

What are your options to reduce your risk?

The following things can be done to reduce your risk of developing breast cancer:

Screening – [insert here]
Lifestyle change – [insert here].
Medication for preventions – [insert here]

In summary

Since your first visit to the clinic your risk has increased because of a high SNP score and moderately high breast density.

We are very grateful for your help with the FH-Risk studies. In turn we hope that you feel they have helped you.

With best regards,

Example of PRS/SNP analogy women received

We have found over 300 places along your DNA which are related to your risk of breast cancer (These are called single nucleotide polymorphisms or SNPs (pronounced SNIPS) for short. We have measured all of these sites in your blood sample. At some of the 300 places there is a change which results in a small increase risk of breast cancer which we call A for the sake of illustration. Changes at other places may result in a small decrease in risk which we call B. If you have more As than Bs you are at overall increased risk. If you have more Bs than As you are at decreased risk. Most women have about the same number of As and Bs so there is little change in risk. Others may have more of one than the other, which can result in markedly higher or lower risk. We add these results to your standard risk factors and your breast density to come up with an overall risk score as will be outlined in our letter to you. Putting SNPs together with all the other risk factors helps us give a more precise risk score for you which helps decide your continued need for screening and whether to suggest preventive therapy. So with all this in mind, we have found that you have more As than Bs, meaning that your risk has slightly increased on this point.

Study questionnaire:

Your updated breast cancer risk score:

Your views

We would appreciate your views on breast cancer risk and on your most recent consultation with the Family History Risk and Prevention Clinic [redacted] As you know, through our research we have learnt more about how to calculate breast cancer risk. As a result, risk scores for some women may have changed. Completion of this survey will help us to improve the service for people like you.

This survey is made up of **15 questions** and should take no more than 10 minutes to complete.

All information will be treated as strictly confidential.

If you have any questions about the survey or have any concerns about your

Study ID:

Please turn over →

1. Please select **ONE** of the statements below that best describes your breast cancer risk in relation to other women of a similar age *(please tick only **ONE** box)*.

☐ I believe my risk of developing breast cancer in the next 10 years is **much higher** than other women of my age.

☐ I believe my risk of developing breast cancer in the next 10 years is **a bit higher** than other women of my age.

☐ I believe my risk of developing breast cancer in the next 10 years is **about the same** as other women of my age.

☐ I believe my risk of developing breast cancer in the next 10 years is **a bit lower** than other women of my age.

☐ I believe my risk of developing breast cancer in the next 10 years is **much lower** than other women of my age.

2. Please read the statement and items below and select the option that best describes your breast cancer risk today *(please tick only **ONE** box)*.

I believe my percentage risk of developing breast cancer in my lifetime is…

☐	☐	☐
30% or greater<br>(1 in 3)	Between 17% and 29.9%<br>(1 in 4-6)	Less than 17%<br>(less than 1 in 6)

3. Since your last consultation with the Family History Risk and Prevention Clinic [redacted] which of the following statements best describes how you view your breast cancer risk today *(please tick only **ONE** box).*

My risk of breast cancer has **increased a lot**. ☐

My risk of breast cancer has **increased a little**. ☐

My risk of breast cancer has **stayed the same**. ☐

My risk of breast cancer has **decreased a little**. ☐

My risk of breast cancer has **decreased a lot**. ☐

I do not know what my risk status currently is. ☐

4. Which risk factors do you believe were included in your updated breast cancer risk that were not included before *(for each risk factor below please tick only **ONE** box).*

Age

☐ New risk factor	☐ Included before	☐ Not a factor included in my updated risk	☐ Not sure

Breast density (the ratio of fibroglandualar tissue to fat in the breast)

☐ New risk factor	☐ Included before	☐ Not a factor included in my updated risk	☐ Not sure

Relatives affected by breast cancer (family history)

☐ New risk factor	☐ Included before	☐ Not a factor included in my updated risk	☐ Not sure

Breast cancer gene testing (for example BRCA)

☐ New risk factor	☐ Included before	☐ Not a factor included in my updated risk	☐ Not sure

BMI (body mass index)

☐ New risk factor	☐ Included before	☐ Not a factor included in my updated risk	☐ Not sure

Single Nucleotide Polymorphisms (SNPs, alterations in the DNA associated with breast cancer)

☐ New risk factor	☐ Included before	☐ Not a factor included in my updated risk	☐ Not sure

Please turn over →

5. Thinking about your last consultation at the Family History Risk and Prevention Clinic [redacted] please read each statement below and select the option that best indicates how you feel about the information you received *(please tick only **ONE** box per statement).*

I feel well informed about my breast cancer risk.

☐	☐	☐	☐	☐	☐	☐
Strongly agree	Agree	Somewhat agree	Undecided	Disagree Somewhat	Disagree	Strongly disagree

I feel satisfied with the amount of information I have been given.

☐	☐	☐	☐	☐	☐	☐
Strongly agree	Agree	Somewhat agree	Undecided	Disagree somewhat	Disagree	Strongly disagree

I am confused by the information I have been given.

☐	☐	☐	☐	☐	☐	☐
Strongly agree	Agree	Somewhat agree	Undecided	Disagree somewhat	Disagree	Strongly disagree

The information was clear.

☐	☐	☐	☐	☐	☐	☐
Strongly agree	Agree	Somewhat agree	Undecided	Disagree Somewhat	Disagree	Strongly disagree

6. Please read the statement and items below and select the option that best describes your current level of worry about getting breast cancer someday *(please tick only **ONE** box)*.

How often do you worry about developing breast cancer?

☐	☐	☐	☐
Never	Rarely	Sometimes	Almost all the time

7. Please let us know if there is anything important to you that we have not asked about regarding your breast cancer risk below:

Thank-you for completing this survey.

Please return your completed questionnaire to the team in the pre-paid envelope provided together with a copy of your signed consent form.
